# Clinicopathologic correlations of renal microthrombosis and inflammatory markers in proliferative lupus nephritis

**DOI:** 10.1186/ar3856

**Published:** 2012-05-28

**Authors:** Elena Gonzalo, Oscar Toldos, María P Martínez-Vidal, María C Ordoñez, Begoña Santiago, Antonio Fernández-Nebro, Estíbaliz Loza, Isabel García, Myriam León, José L Pablos, María Galindo

**Affiliations:** 1 Instituto de Investigación Hospital 12 de Octubre (i+12), Avenida de Córdoba sn, 28041 Madrid, Spain; 2 Servicio de Anatomía Patológica, Hospital 12 de Octubre, Avenida de Córdoba sn, 28041 Madrid, Spain; 3 Servicio de Reumatología, Hospital 12 de Octubre, Avenida de Córdoba sn, 28041 Madrid, Spain; 4 Servicio de Reumatología, Hospital Regional Universitario Carlos Haya, Avenida Carlos Haya sn, 29010 Málaga, Spain; 5 Unidad de Investigación de la Fundación Española de Reumatología, Sociedad Española de Reumatología, Calle Marqués del Duero 5, 1°, 28001 Madrid, Spain; 6 Servicio de Anatomía Patológica, Hospital Regional Universitario Carlos Haya, Avenida Carlos Haya sn, 29010 Málaga, Spain

## Abstract

**Introduction:**

Microthrombosis is often observed in lupus nephritis (LN) lesions, but its clinical significance is unknown. We evaluated the clinicopathologic correlations of renal microthrombosis and inflammatory markers in LN.

**Methods:**

Kidney biopsies from 58 patients with systemic lupus erythematosus (SLE) proliferative nephritis were analyzed with immunohistochemistry (IHC) for intravascular platelet aggregates (CD61), macrophagic infiltration (CD68), and activated complement deposition (C4d). Clinical data at the time of kidney biopsy and follow-up were analyzed with regard to pathologic IHC data.

**Results:**

Microthrombosis was present in 52% of the tissues. It was significantly more prevalent in patients with antiphospholipid antibodies (aPLs) (62% versus 42%). The presence of microthrombosis significantly correlated with higher macrophagic infiltration. Macrophagic infiltration but not microthrombosis was significantly correlated with C4d deposition. Only macrophagic infiltration showed a correlation with SLE and renal activity (proteinuria and active sediment), whereas neither the presence of CD61^+ ^microthrombi nor the extent of C4d deposition correlated with LN severity or outcome.

**Conclusions:**

Microthrombosis is associated with higher macrophagic infiltration in LN but does not seem to increase independently the severity of renal damage. Macrophagic infiltration was the best marker of SLE and renal activity in this LN series.

## Introduction

Lupus nephritis (LN) develops in 30% to 50% of patients with systemic lupus erythematosus (SLE) [1]. Standard clinical practice is to perform a renal biopsy if clinical or analytic parameters suggest renal involvement. Although some clinical variables, such as elevation of serum creatinine or persistent elevations of blood pressure, have prognostic value, histologic information obtained from biopsies continues to be indispensable for classification and outcome prediction [2,3]. Among local inflammatory markers, glomerular and interstitial macrophage accumulation is a feature of the most aggressive forms of human glomerulonephritis [4]. These cells, together with dendritic cells, are the major source of inflammatory cytokines [5], and their interaction with resident T cells may amplify renal inflammation. Monocyte numbers in the urine and urinary levels of the monocyte chemoattractant protein-1 (MCP-1) have been found to be useful markers to monitor the activity of LN [6,7]. In patients with proliferative forms of LN, MCP-1 also may play a role in modulating interstitial inflammatory cell infiltration and tubulointerstitial damage via the transforming growth factor (TGF)-beta 1 pathway [8]. Therefore, glomerular and tubular macrophagic infiltration has important pathogenetic implications, and it is one of the individual variables that best correlates with clinical parameters [9].

Vascular occlusions can be observed in LN and may relate to worse renal outcomes [10]. Acute thrombosis or chronic occlusive lesions in glomeruli and/or renal arterioles have also been described in patients with antiphospholipid syndrome (APS) [11,12]. Acute thrombosis in LN, as detected with routine histology, is rarely observed, possibly because of its relatively sparse presence and the small size of tissue samples obtained with renal biopsy. Other chronic, nonspecific vascular occlusive histologic lesions may relate to older age and cardiovascular risk factors associated with arteriosclerotic disease, even in patients without aPLs (antiphospholipid antibodies) [13]. This type of chronic occlusive vascular lesions has been associated with a worse renal-function outcome. Immunohistochemical detection of CD61^+ ^intravascular platelets in SLE renal biopsies increases the sensitivity to detect microthrombosis and reveals a high prevalence of microthrombotic lesions in either aPL-negative or -positive LN patients [13]. Whether microthrombotic lesions are a consequence of renal inflammation or independently contribute to renal damage is unclear [10-12].

A link between activation of the complement system and aPL-induced thrombosis has been described in murine APS models [14,15]. In SLE patients with thrombosis and vascular aPL-related pathology, increased C4d deposition on platelets has been reported [16]. In SLE patients without aPL, C4d kidney deposition also correlates with thrombosis [17]. Therefore, complement activation might be a common mechanism of renal inflammation and thrombosis in patients with LN with or without aPL.

We undertook this study to investigate the potential relation between local complement activation, macrophagic infiltration, and renal microthrombosis, as well as their potential independent contribution to renal damage in a large series of SLE patients with proliferative nephritis. Our data provide evidence of the relation between these three processes but do not support an independent contribution of microthrombosis to renal damage.

## Materials and methods

### Patients and kidney biopsies

From a large cohort of SLE patients followed up at two University Hospitals between 1986 and 2010, we selected those with available kidney biopsy specimens to perform histologic and immunohistochemical studies. All patients fulfilled at least four of the American College of Rheumatology criteria for the diagnosis of SLE [18]. The study received approval by the Ethics Committee of Hospital 12 de Octubre. This study was performed on previously collected and filed pathology materials obtained after informed patient consent. Kidney biopsies had been performed for diagnostic purposes in patients with proteinuria higher than 500 mg/day, abnormal urinary sediment, or elevated serum creatinine level. We considered that clinicopathologic correlations could be independently analyzed in different episodes, and therefore, biopsies from different episodes of the same patient were included when available. Kidney histology was evaluated and classified according to the ISN/RPS classification [19] by a pathologist who was blinded to patients' clinical and IHC data. Hematoxylin-eosin and phosphotungstic acid-hematoxylin (PAS) stained sections were evaluated. The whole series included 86 patients. In total, 101 histologic samples were analyzed. Among them, we selected 65 (64%) samples with proliferative nephritis (ISN/RPS types II, III, and IV) from 58 patients for further study. Their demographic, clinical, laboratory, and pathology characteristics before and at renal biopsy are detailed in Table 1.

**Table 1:** Demographic, clinical, and laboratory characteristics of SLE patients with proliferative nephritis

	Patients (*n *= 58)
	*n *(% or range)
Sex: F/M	54/4 (93/7)
Mean age (years)	39 ± 12 (22-75)
Mean age at SLE diagnosis (years)	26 ± 11 (8-66)
Mean age at nephritis (years)	29 ± 12 (8-73)
Mean duration of follow-up (months)	97 (1-273)
Cardiovascular risk factors	35 (58)
Positive aPL	31 (53.4)
APS	8 (14.8)
ANA (+)	58 (100)
a-DNA (+)	49 (84.5)
a-Ro (+)	21 (42.9)
a-Sm (+)	16 (33.3)
a-RNP (+)	11 (22.9)
Low complement levels	52 (91.2)
Treatment before first renal biopsy	
Antiaggregation	20/65 (31)
Oral anticoagulation	2/65 (3.1)
Glucocorticoids	50/65 (77)
≤7.5 mg/d prednisone	42%
< 7.5 to 30 mg/d prednisone	43%
> 30 to 60 mg/d prednisone	10%
> 1 mg/kg/d prednisone	5%
Azathioprine	13/65 (20)
Antimalarials	20/65 (31)
ISN/RPS nephritis classification:	
Type II	2/65 (3)
Type III-A	13/65 (20)
Type III-A/C	4/65 (6.1)
Type III-A + V	2/65 (3)
Type IV-SA	10/65 (15.4)
Type IV-SA/C	4/65 (6.1)
Type IV-GA	23/65 (35.4)
Type IV-GA/C	5/65 (7.7)
Type IV-GA/C + V	2/65 (3)
24 hours proteinuria (mg)^a^	3,355.1 ± 3,372.3 (230-19,700)
Hematuria (+)^a^	56 (86.2)
Leukocyturia (+)^a^	52 (80)
Cellular casts (+)^a^	42 (64.6)
Mean serum creatinine (mg/dl)^a^	1.1 ± 0.5 (0.4-3.4)
Mean creatinine clearance (ml/min)^a^	91.8 ± 44.4 (25-289)
Renal failure^a^	17 (26.2)
HBP^a^	35 (53.8)
SLEDAI^a^	20.4 (4-36)
Response to treatment	50 (83.3)
Mean time of response (months)	15 ± 19 (1-101)

Cardiovascular risk factors include high blood pressure (HBP), diabetes, hypercholesterolemia and/or hypertriglyceridemia, smoking, and hormonal contraception. ^a^Values at renal biopsy.

APS was diagnosed according to the Sapporo criteria [20]. Definite APS was considered if at least one of the following clinical and laboratory criteria was satisfied. Clinical criteria included the presence of either vascular thrombosis or pregnancy morbidity. Laboratory criteria included the presence of aPL on two or more occasions at least 6 weeks apart, as demonstrated by one or more of the following: IgG and/or IgM anticardiolipin (aCL) antibodies in moderate or high titer and lupus anticoagulant (LA) activity. LA was detected after updated guidelines of the Subcommittee for the Standardization of Lupus Anticoagulant of the International Society of Thrombosis and Haemostasis [21], whereas aCLs were detected with enzyme-linked immunosorbent assay (ELISA) by following the manufacturer's instructions (Quanta Lite ACA IgG III and IgM III; Inova Diagnostics Inc, San Diego, CA, USA).

The following demographic data were recorded: sex, age, age at SLE diagnosis, age at renal disease, and time from kidney biopsy to the end of follow-up. Clinical and laboratory SLE data, including the disease-activity index SLEDAI [22], cardiovascular risk factors (high blood pressure (HBP), diabetes, hypercholesterolemia and/or hypertriglyceridemia, smoking, and hormonal contraception), APS criteria, and previous therapy were recorded.

Renal manifestations at biopsy and during the follow-up, specific therapy, response and time to response, and relapse and time to relapse were analyzed. Renal manifestations included HBP, renal failure defined as serum creatinine ≥1.5 mg/dl and/or creatinine clearance ≤65 ml/min, 24-hour proteinuria, hematuria, leukocyturia, and cellular casts. Complete clinical response to therapy was defined by 24 hours of proteinuria lower than 500 mg, normal urine sediment, and normal renal function (serum creatinine, < 1.5 mg/dl, and creatinine clearance, > 75 ml/min). Partial response to treatment was defined by improvement in all these parameters not reaching complete-response criteria. The presence of aPL (LA, IgG, or IgM aCL) and APS criteria before and after kidney disease was also recorded.

### Immunohistochemical detection of CD61-positive platelet microthrombi, CD68-positive macrophages, and activated complement factor C4d

Immunohistochemical studies were performed on paraffin-embedded kidney sections. Immunostaining with either monoclonal mouse anti-human CD68 (CD68, clone KP1; DakoCytomation Denmark A/S, Glostrup, Denmark), monoclonal mouse anti-human gpIIIa (CD61, sz21; Immunotech, Marseille, France), or polyclonal rabbit anti-human C4d (C4dpAb; Biomedica Medizinprodukte GmbH & Co KG, Vienna, Austria) were performed. Renal sections from patients with primary thrombotic microangiopathy (TMA) were used as positive controls for CD61. Renal sections from a normal kidney were used as negative controls.

Sections were deparaffinized and pretreated with 10 μg/ml proteinase K (P6556; Sigma-Aldrich Quimica SA, Madrid, Spain) in the case of CD61 detection, or boiled in 1 m*M *EDTA or citrate buffer 10 m*M*, pH 6, for CD68 and C4d detection, respectively. The slides were incubated overnight at 4°C with primary antibodies. Immunohistochemical staining was performed with a peroxidase avidin-biotin complex technique (SK-4100; Vector Laboratories Inc., Burlingame, CA, USA). Diaminobenzidine was used as chromogen substrate, and sections were counterstained with hematoxylin.

Glomerular and extraglomerular CD68 and C4d staining was quantified by using the public domain image-processing program, Image J software [23]. In brief, the percentage of stained area in 10 glomeruli and 10 extraglomerular areas randomly selected from each sample was calculated. Two blinded and independent observers analyzed all slides.

### Statistical analysis

The associations between categoric variables were tested by using the χ^2 ^or Fisher Exact test, where appropriate. The odds ratios (ORs) with the corresponding 95% confidence intervals (95% CIs) were calculated. For continuous variables, the comparisons were carried out by using the *t *test for two independent samples. A Spearman rank correlation was used to detect correlations among different study parameters. *P *values less than 0.05 were considered significant. The analysis was performed by using advanced SPSS software version 15.

## Results

### Histologic and IHC detection of microthrombotic lesions, macrophagic infiltration, and C4d deposition in kidney biopsies

**Figure 1:**
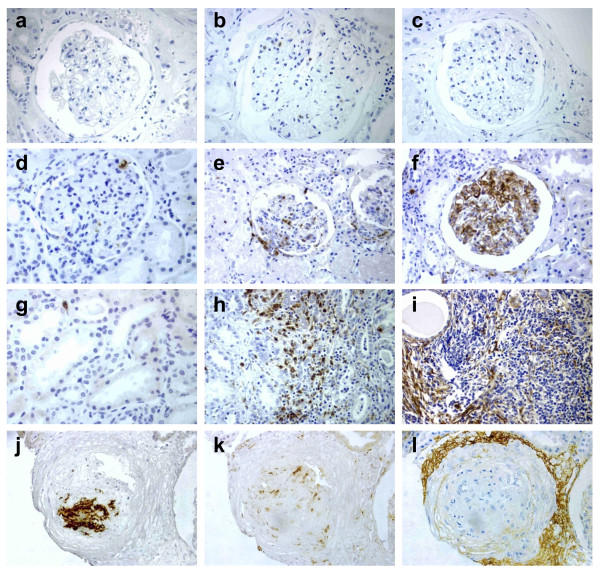
**Immunohistochemical detection of platelet microthrombi lesions, macrophagic infiltration, and activated complement deposition in kidney biopsies**. CD61 (left), CD68 (middle), and C4d (right) antigens were detected with immunoperoxidase (brown) in serial sections of healthy kidney **(a **through **c) **and SLE nephritis tissues **(d **through **l)**. SLE glomerular **(d **through **f) **or extraglomerular **(g **through **i) **markers are shown. Positive CD61 microthrombi **(j)**, CD68 infiltration **(k)**, and C4d perivascular deposition **(l) **were detected. Sections were counterstained with hematoxylin. Original magnification, ×400.

In healthy, histologically normal kidney sections used as controls for IHC, CD61-positive platelet aggregates were not detected (Figure 1a). CD61-positive microthrombi were detected in 52% of the proliferative LN tissues (79% in glomerular and 62% in extraglomerular vessels) (Figure 1d, g, j).

CD68-positive macrophages were identified in the mesangial areas of both normal (Figure 1b) and lupus glomeruli (Figure 1e), as well as in extraglomerular areas (Figure 1h). The density of CD68-positive macrophages in both glomerular and extraglomerular areas was greatly increased in lupus patients. The mean stained area was 2.37% ± 3.02% and 0.72% ± 0.98% in glomerular and extraglomerular regions of lupus sections, respectively, compared with 0.0004% ± 0.0005% and 0.0003% ± 0.0007% in normal kidney.

C4d deposition was undetectable in normal kidney sections (Figure 1c), whereas it was detected in most SLE biopsies, in either glomerular (78%) or extraglomerular (86%) regions (Figure 1f, i). The mean C4d-stained glomerular and extraglomerular areas were 2.76% ± 3.73% and 1.04% ± 1.32%, respectively. The main extraglomerular pattern was diffuse linear C4d deposition along peritubular capillaries (98%), although some focal arteriolar C4d staining was also found in interstitial vessels (19%) (Figure 1l).

### Clinical and laboratory correlations of platelet aggregates, macrophage infiltration, and C4d deposition

Patients with glomerular CD61-positive microthrombi had significantly higher glomerular (*P *= 0.03) macrophage infiltration. C4d extraglomerular deposition positively correlated with extraglomerular macrophage infiltration (*r *= 0.4; *P *= 0.004), but mean C4d deposition was similar in patients with or without CD61-positive microthrombi.

The presence of platelet aggregates was significantly higher in patients with positive aPL (62% versus 42%; OR, 4.4; CI, 1.5 to 12.8; *P *= 0.005), but it was similar in patient groups with or without definite APS or with or without previous cardiovascular risk factors. No correlations were found between microthrombosis and SLE or renal clinical data, including positive anti-dsDNA antibodies, low complement levels, SLEDAI, 24-hour proteinuria, serum creatinine, creatinine clearance, or the presence of hematuria, leukocyturia, cellular casts, HBP, or renal failure.

Both glomerular and extraglomerular macrophagic infiltrations positively correlated with SLE manifestations. Glomerular macrophagic infiltration correlated with SLEDAI (*r *= 0.5; *P *< 0.001) and was significantly higher in patients with positive anti-dsDNA antibodies (*P *< 0.001) or low complement levels (*P *< 0.001) at biopsy. Regarding renal manifestations, glomerular macrophagic infiltration positively correlated with 24-hour proteinuria (*r *= 0.4; *P *= 0.002) and was associated with the presence of microhematuria (*P *< 0.001), and cellular casts (*P *= 0.001). Extraglomerular macrophage infiltration correlated with SLEDAI (*r *= 0.3; *P *= 0.01) but was not associated with the presence of anti-dsDNA antibodies or low complement levels. Regarding renal manifestations, extraglomerular macrophage infiltration correlated with 24-hour proteinuria (*r *= 0.4; *P *= 0.001) but not with other analytic parameters. No correlation was found between glomerular or extraglomerular macrophage infiltration and renal function defined by creatinine clearance at biopsy.

Extraglomerular C4d deposition was significantly higher in patients with positive anti-dsDNA antibodies (*P *= 0.01) and correlated with SLEDAI (*r *= 0.4; *P *= 0.003) and 24-hour proteinuria (*r *= 0.3; *P *= 0.03). No differences in extraglomerular C4d deposition were found when we considered other clinical or laboratory parameters of renal function and SLE activity. Glomerular C4d deposition did not show any specific trend stratified by all clinical and laboratory variables. Clinical and laboratory correlations of platelet aggregates, macrophage infiltration, and C4d deposition are detailed in Table 2.

**Table 2:** Clinical and laboratory associations of CD61-positive microthrombi, CD68-positive macrophage infiltration, and C4d deposition

		CD61 microthrombi		CD68^+ ^infiltration		C4d deposition	
		**+**	**-**	**Glomerular**	**Extraglomerular**	**Glomerular**	**Extraglomerular**

SLEDAI		20.5 ± 6.9	20.3 ± 6.6	*r *= 0.5^a^	*r *= 0.3^a^	*r *= -0.1	*r *= 0.4^a^

aPL	+	69%	33%	1.9 ± 1.9	1 ± 1.3	2.6 ± 2.9	0.9 ± 1.4
	-	31%	67%	2.1 ± 3.1	0.4 ± 0.6	2.9 ± 4.7	1 ± 1.2

anti-dsDNA	+	91%	74%	2.8 ± 3.2^a^	0.8 ± 1	2.4 ± 2.9	1.2 ± 1.4^a^
	-	9%	26%	0.6 ± 0.7	0.5 ± 0.8	3.8 ± 6	0.5 ± 0.5

Low complement	+	94%	87%	2.6 ± 3.1^a^	0.7 ± 1	2.4 ± 2.9	1.1 ± 1.4
	-	6%	13%	0.5 ± 0.5	0.9 ± 0.9	5.4 ± 8	0.8 ± 0.5

24-hour proteinuria (g)		3.2 ± 3.1	3.6 ± 3.6	*r *= 0.4^a^	*r *= 0.4^a^	*r *= 0.1	*r *= 0.3^a^

Hematuria	+	88%	84%	2.7 ± 3.2^a^	0.8 ± 0.9	2.7 ± 3.9	1.1 ± 1.4
	-	12%	16%	0.6 ± 0.7	0.6 ± 1.4	2.9 ± 2.9	0.5 ± 0.6

Leukocyturia	+	76.50%	84%	2.7 ± 3.2^a^	0.7 ± 0.9	2.7 ± 3.6	1.1 ± 1.4
	-	23.50%	16%	1.3 ± 1.5	0.7 ± 1.2	2.8 ± 4.5	0.8 ± 1

Cellular casts	+	62%	68%	3.1 ± 3.5^a^	0.8 ± 0.8	3.1 ± 4.2	1.1 ± 1.2
	-	38%	32%	1.1 ± 1.3	0.7 ± 1.3	2.1 ± 2.7	0.9 ± 1.6

Creatinine clearance		97.9 ± 50.2	84.3 ± 35.6	*r *= -0.01	*r *= -0.2	*r *= 0.1	*r *= - 0.1

Response to treatment	+	81%	95%	3.1 ± 3.5	0.8 ± 0.9	2.9 ± 4.2	1.2 ± 1.5
	-	19%	5%	2.2 ± 1.4	1.3 ± 1.7	1.9 ± 1.5	0.9 ± 0.4

Mean time to response		25.1 ± 27.9	8.8 ± 10.8	*r *= -0.15	*r *= -0.26	*r *= -0.17	*r *= -0.05

LN relapse	+	33%	45%	2.8 ± 3.3	0.6 ± 0.7	1.9 ± 2.9	1.1 ± 1.1
	-	67%	55%	3.4 ± 4.4	0.7 ± 0.8	2.9 ± 3.5	1.4 ± 1.9

Renal failure at 1 year	+	17.40%	15%	3.2 ± 3.6	0.8 ± 0.9	2.9 ± 4.2	1.3 ± 1.6
	-	82.60%	85%	1.9 ± 2.1	1.5 ± 1.6	1.3 ± 1.7	1.2 ± 0.7

Renal failure at last visit	+	20.80%	30%	3.3 ± 3.7	0.9 ± 1	3 ± 4.4	1.3 ± 1.6
	-	79.20%	70%	2.1 ± 1.9	1 ± 1.4	1.5 ± 1.7	1 ± 0.7

^a^*P *< 0.05.

As initial therapy for LN, all patients received high doses of steroids (≥1 mg/kg/day) as well as one of the following immunosuppressive drugs: cyclophosphamide (79%), mycophenolate mofetil (17.7%), or azathioprine (1.6%). In addition, 54.2% received aspirin, and 5.1%, oral anticoagulants. Most patients (77%) had been treated with steroids before LN development for other manifestations at variable doses (Table 1). Twenty patients were treated with low-dose aspirin, and two patients were taking oral anticoagulation before LN development. In most patients treated with aspirin, indication was positive for LA and/or aCL antibodies (*n *= 12). Some patients received aspirin for HBP. The two patients receiving oral anticoagulation had previous venous or arterial thrombosis. Different groups of patients stratified by aspirin or steroid dose did not show any trend toward a different expression of LN or platelet aggregates incidence, or differences in the long-term outcomes. Fifty (83.3%) patients responded to treatment, and the mean time to partial or complete response was of 15.2 months (range, 1 to 101 months). To analyze the potential long-term effect of immunohistochemical findings on clinical and renal outcomes, we selected only patients homogeneously treated with intravenous cyclophosphamide according to the NIH protocol after excluding repeated episodes of LN [24]. In brief, induction therapy consisted of six monthly boluses of intravenous cyclophosphamide (0.5 to 1 g per square meter, further adjusted by leukocyte count) and glucocorticoids. After 6 months, patients were treated with intravenous cyclophosphamide boluses every 3 months and for at least 12 months after remission or stability. Maintenance therapy was variable and, according to clinical practice, included azathioprine, mycophenolate mofetil, or antimalarials with glucocorticoids. Taking into account only those patients (*n *= 43) who had received the same induction treatment, we did not found significant correlations between the presence of CD61^+ ^microthrombi, macrophagic infiltration, or C4d deposition, and clinical outcomes including percentage of response to therapy, mean time to response, percentage of relapse, and long-term renal function.

## Discussion

These data show that renal microthrombosis is highly prevalent in LN, affecting half of the patients with proliferative LN. It is more prevalent in patients with aPL, consistent with previous reports [13]. Moreover, microthrombosis correlates with higher macrophagic infiltration as a marker of inflammatory activity but not with C4d deposition. However, only macrophagic infiltration showed a correlation with SLE renal activity (proteinuria and active sediment), and neither the presence of CD61^+ ^microthrombi nor C4d deposition correlated with LN severity or outcome. Although the mechanisms of thrombosis may be different, previous studies point to complement activation and inflammation as common mechanisms in LN- and aPL-associated vascular pathology. In APS, a "two hits" theory has been proposed, in which the presence of aPL would lead to thrombophilia through endothelial cell, platelet, and monocyte activation ("first hit"), and a "second hit" related to inflammation (that is, Toll-like receptors and complement activation, in response to microbial agents, might synergize and result in clotting events [25]. Our data link inflammatory infiltration and thrombosis in LN but are less clear regarding the relation of complement activation and thrombosis.

Several studies point to the involvement of complement in the pathogenesis of APS. In animal models, complement factors C3 and C5 are essential in aPL-mediated pathology [15]. In these models, endothelial cell activation and inflammatory cell infiltration around blood vessels are observed in placental lesions, suggesting that complications of pregnancy are primarily due to inflammation [14]. In humans, a few studies also point to complement activation in aPL-mediated thrombosis [26-28]. Sera from patients with positive aPL enhance complement fixation on platelets, which is associated with arterial thrombosis [16]. Platelet-associated C4d has been reported in 18% of patients with SLE, particularly in those with aPL [29]. In addition, hypocomplementemia and increased plasma levels of anaphylotoxins C3a and C4a have been described in primary APS [26]. However, a direct correlation between increased levels of anaphylotoxins and clinical thrombosis has not been demonstrated [26,28].

Specifically in LN, immune-mediated vascular injury may predispose to platelet accumulation and *in situ *complement activation. Intense glomerular C4d deposition has been previously found associated with renal microthrombi in LN patients with or without aPL [15,28]. However, because both processes may be associated with proliferative LN and higher activity indexes, this may be an indirect association [26]. We have specifically analyzed patients with proliferative LN and considered macrophage infiltration as an additional marker of inflammatory activity. Our data do not demonstrate a direct relation between C4d deposition and microthrombosis. Instead, C4d deposition correlated with the intensity of macrophagic infiltration, which in turn was associated with microthrombosis. Deposition of C4d in peritubular capillaries reflected SLE activity, consistent with previous reports [30,31]. Neither C4d nor microthrombi were directly related to the severity or outcome of LN.

The detection of CD61 intravascular platelets with IHC is far more sensitive to acute microthrombi in patients with LN compared with routine histologic methods. Thrombi detected with regular histology might have a different impact on renal outcomes but are rarely observed. Clinical observations support that TMA in APS or SLE nephritis has an important impact on renal function and requires specific therapy. However, TMA may occur independent of SLE nephritis, and its prevalence associated with LN is very low. Only two cases had microthrombi detected by histology in our series, precluding analyses of the clinical and subclinical (immunohistochemical) correlations in LN. Our data do not support any difference in renal manifestations and outcomes at 6 and 12 months, and further follow-up after therapy in patients with IHC detected CD61-positive microthrombi. The retrospective design of our study limits the interpretation of results regarding clinical correlations and prognosis. Microthrombi detected with IHC may have a lower impact on renal function and outcome than do larger histologic microthrombi. Longitudinal prospective studies, including patients with LN and repeated biopsies after induction treatment, will help us to better define the real significance of this kind of histologic and IHC lesion.

Macrophagic infiltration was the best marker of renal impairment at kidney biopsy, consistent with previous studies [32]. Glomerular and interstitial macrophage infiltration is a feature of the more aggressive forms of LN and the individual variable that best correlates with clinical parameters, including SLE activity and renal manifestations. We found a correlation with activity measures, including the level of proteinuria and active sediment, but not with renal function at biopsy or follow-up. A previous study showed that the persistence of glomerular and extraglomerular macrophagic infiltrates after therapy correlates with long-term renal-function outcomes (doubling of serum creatinine and end-stage renal disease) [32]. However, neither these nor our data demonstrates predictive value for the degree of macrophage infiltration at first biopsy and renal-function outcomes.

## Conclusions

In summary, our results show a relation between renal microthrombi and local inflammatory cells infiltration in patients with LN. Renal macrophagic infiltration was the best marker of SLE and renal clinical activity, whereas microthrombi or C4d deposition did not have a direct correlate with severity or prognosis in LN.

## Abbreviations

aCL: anticardiolipin antibodies; aPLs: antiphospholipid antibodies; APS: antiphospholipid syndrome; ELISA: enzyme-linked immunosorbent assay; HBP: high blood pressure; IHC: immunohistochemistry; ISN/RPS: international society of nephrology/renal pathology society; LA: lupus anticoagulant; LN: lupus nephritis; MCP-1: monocyte chemoattractant protein-1; NIH: national institute of health; OR: odds ratio; PAS: phosphotungstic acid-hematoxylin; SLE: systemic lupus erythematosus; TGF-beta 1: transforming growth factor-beta 1; TMA: thombotic microangiopathy; SLEDAI: systemic lupus erythematosus disease activity index.

## Competing interests

The authors declare that they have no competing interests.

## Authors' contributions

EG and BS carried out IHC analyses. OT, IG, and ML carried out histologic studies at Hospital 12 de Octubre (OT) and Hospital Regional Carlos Haya (IG, ML), respectively. MPM, MG, MCO, and AFN participated in collecting and analyzing clinical data, both at Hospital 12 de Octubre (MPM, MG) and at Hospital Regional Carlos Haya (MCO, AF), respectively. EL participated in the design and statistical analysis. JLP and MG participated in the conception and design of the study, coordination, and analysis of data. All authors participated in drafting and revising the manuscript and read and approved its final version.
